# Objective vision screening using PlusoptiX for children aged 3–11 years in rural Turkey

**DOI:** 10.1186/s12886-019-1080-7

**Published:** 2019-03-12

**Authors:** Silay Canturk Ugurbas, Numan Kucuk, Irem Isik, Atilla Alpay, Cagatay Buyukuysal, Suat Hayri Ugurbas

**Affiliations:** 1 Department of Ophthalmology, Faculty of Medicine, Zonguldak Bulent Ecevit University, Esenkoy-Kozlu, 67600 Zonguldak, Turkey; 2 Department of Biostatistics, Faculty of Medicine, Zonguldak Bulent Ecevit University, Esenkoy-Kozlu, Zonguldak, 67600 Turkey

**Keywords:** Amblyopia, Vision screening, Amblyopia risk factors, Plusoptix, Photoscreening

## Abstract

**Background:**

This population based cross sectional study was conducted to detect amblyopia risk factors and myopia in a rural district of Northwestern Turkey by using PlusoptiX S12R (Photoscreener PlusoptiX Inc., Nuremberg, Germany).

**Methods:**

Children from 38 rural schools in Caycuma district of Zonguldak Turkey underwent vision screening in their school using PlusoptiX S12 photoscreener. Data were analyzed using the factory default level 5 referral criteria targeting 80% sensitivity and 95% specificity. Referral, unable readings, and positive predictive value (PPV) were reported.

**Results:**

Data from 2846 children were analyzed. Mean age was 7.9 years (±0.8) (range 36 months to 11 years). Three hundred ten (11%) were referred of whom 32% were read as ‘unable’. 150 children (48% of the referred) received a gold standard examination. Positive predictive value of PlusoptiX was 69%. PPV was 83% when unable readings were excluded. 93 children with amblyopia risk factors were identified. Only 26% (*n* = 25) had received glasses priorly. 49 children had amblyopia of whom 33 were newly diagnosed.

**Conclusions:**

PlusoptiX showed a reasonable level of positive predictive value in community setting and the device could be a useful tool for vision screening in preschoolers and schoolers. We found most of the amblyogenic refractive errors were underdiagnosed in rural school children leading to a call for action on vision screening.

## Introduction

Undiagnosed and untreated vision deficits in children can affect a child’s school performance and life time well-being. Amblyopia is the most frequent cause of monocular visual impairment in children and is defined as reduced best- corrected visual acuity in the absence of organic abnormalities accompanied by one or more known amblyopia risk factors, such as strabismus, anisometropia, isoametropia, and cataract [1, 2]. Amblyopia with an estimated prevalence of 2–5% meets all criteria for a World Health Organization screening program based on benefits of diagnosis and treatment [3, 4]. Early vision testing (0–6 years) and diagnosis guarantees faster treatment and improvement of amblyopia [5]. However, the critical period for visual development may extend up to 12 years of age [6]. Recent PEDIG (Pediatric Eye Disease Investigator Group) studies also demonstrate that amblyopia treatment should be addressed in children even older than that [7].

Currently there is no consensus on the preferred, validated, and effective vision and amblyopia screening protocol. The PlusoptiX S12 is a photoscreener that uses infrared and wavefront technology to perform binocular non-cycloplegic autorefraction within seconds from a distance of 1 m. Its accuracy to detect myopia, astigmatism, anisometropia, and hyperopia is discussed elsewhere [8–12]. The applicability of the device for vision screening purposes is to detect the risk of having an amblyopia risk factor. A ‘pass’ or ‘refer’ response is generated by the device when threshold criteria for a target refractive error is met. Such criteria is defined by the manufacturer and may be modified by the user according to the desired sensitivity and specificity to be achieved. The photoscreener’s rapid assessment and ability to test a wide age range of children provides opportunity for an alternative to visual acuity tests aiming to detect the same target condition.

Majority of studies using PlusoptiX is carried out in eye clinics where the environment is adjustable for best measurement performance. Studies of its use in real world community setting is limited. The objective of this article to evaluate the results of school photoscreening using the latest version of PlusoptiX in rural northwestern Turkey. To our knowledge this is the first study reporting results of photoscreening in Turkish school children.

## Methods

The present study conducted adhered to the tenets of Declaration of Helsinki and approval was obtained from Bulent Ecevit University clinical studies ethics committee to collect and review data on screening performed in 2016. Screening was conducted in Caycuma municipiality in collaboration of Zonguldak Governorship who funded the PlusoptiX S12R, Zonguldak Public Health Authority who provided the screening personnel and Bulent Ecevit University Ophthalmology Department who examined the referred children and reviewed the screening images. The screening sessions were performed by two public health nurses. A training session for the nurses were provided at Bulent Ecevit University Eye Clinic. The screening nurses were initially accompanied in two successive sessions at the screening site by the senior investigator. Screening sessions were held five times a week for a period of one month. Parental permission and child assent was obtained ahead of screening. After screening parents were sent a letter indicating the screening outcome and to take their children to an ophthalmologist for a comprehensive eye examination if the result was positive for instrument’s referral criteria (Table 1). Children in glasses at time of screening and children for whom screening measurements could not be completed despite several attempts, were also referred. Name of the physician and contact information of the university eye clinic was provided. Electronic records of screening photographs were analyzed by the senior investigator (SCU).

**Table 1:** The threshold for referral criteria used for the PlusoptiX S12 were the manufacturer’s criteria for 85% sensitivity and 90% specificity on receiver operating characteristic (ROC) 5

Age (months)	Astigmatism (DC)	Myopia (D)	Hypermetropia (D)	Anisometropia (D)	Gaze Asymmetry (degrees)
12–36	≥2.0	≥2.0	≥3.0	≥1	110
36–72	≥1.5	≥1.5	≥2.5	≥1	10
72–300	≥1.5	≥0.75	≥2.5	≥1	10

*D* diopter

### Referral criteria

PlusoptiX can evaluate amblyogenic risk factors based on the acquired non-cycloplegic autorefractor readings. The device triggers a referral if the measurement exceeds a user-defined set of values for anisometropia, hyperopia, astigmatism, and myopia. PlusoptiX S12 referrral criteria can be adjusted to five different levels sensitivity and specificity. The relationship between the sensitivity and specificity for a given screening program for various referral cut-offs is graphically demonstrated by a “Receiver-Operator Characteristic (ROC) Curve. In this study users referred children based on factory default threshold value for ROC 5 curve desired for 80% sensitivity and 95% specificity. The threshold criteria used for referral consists of age dependent threshold values for anisometropia, hyperopia, astigmatism, and myopia and is summarized in Table 1. The device will also automatically refer if unable to obtain a reading greater than − 7.00 D - + 5.00 D range, with pupillary abnormalities or in the presence of significant strabismus measuring larger than 10 degrees. The children referred underwent gold standard cycloplegic eye examination (GSE). The criteria for prescribing glasses according to GSE were hyperopia ≥ + 3.50 D, myopia ≥ − 0.75 D, astigmatism ≥ + 1.50 D and anisometropia ≥ + 1.00 D. The criteria for the diagnosis of an amblyopia risk factor was based on guidelines for automated vision screening: astigmatism > 2.00D, hyperopia > 4. 00D, myopia > − 3.00 D and anisometropia > 2.0 D [13].

Amblyopia was defined as a two line difference in the best corrected visual acuity or in isoametropic cases failure to reach age appropriate visual acuity level with the detected refractive error in spectacles in the absence of ocular pathology. For the purposes of this study minimum of 2 visits were required for children who had not received glasses priorly to determine presence of amblyopia with adequate correction.

## Results

**Fig. 1:**
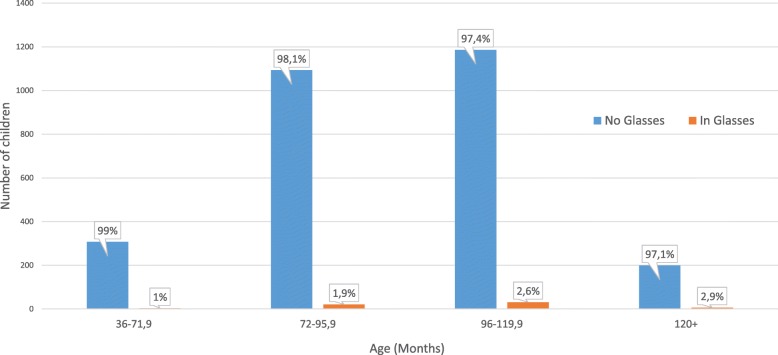
Age Distribution of Screened Children and Use of Spectacles. Bar graph showing the age of children, in months, who underwent a vision screening. The number children in glasses at time of screening and its percentage in different age groups is also shown

Screening sessions were held daily for a period of one month in 38 schools. Data from 2846 children were analyzed. Mean age of children was 7.9 years (±1.4 range 3–11 years). Average spectacle wear was found to be 2%. The age distribution and spectacle wear among age groups is shown in Fig. 1. PlusoptiX referred 11% (*n* = 310) of children of whom 48% (*n* = 150) had GSE. Of the 310 referred 105 were *unable* examinations. Only 5 was due to poor cooperation. 2 readings were considered inconclusive.

**Fig. 2:**
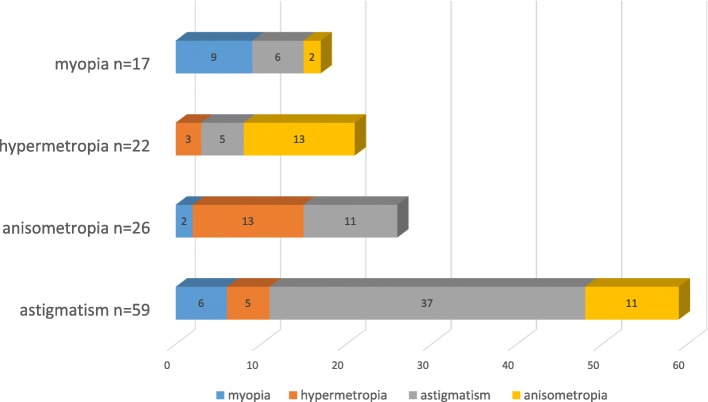
Distribution of types of refractive error in children prescribed glasses in the study population. The above graph shows distribution of refractive errors in children who fulfilled the criteria for needing glasses based on gold standard eye examination (GSE) results. The criteria for prescribing glasses according to GSE were hyperopia ≥ + 3.50 D, myopia ≤ − 0.75 D, astigmatism ≥ + 1.50 D and anisometropia ≥ + 1.00 D. Color coded bars show relative abundance of myopic astigmatism and anisomyopia, hypermetropic astigmatism, anisohyperopia and anisoastigmatism

Of the 150 children who received a GSE, 3 records were omitted due to poor records. 102 children had a positive examination meaning that they met the criteria for prescribing glasses or had strabismus. PPV for diagnosis of positive examination was 69%. A total of 98 children met the criteria for prescribing glasses. There were only four children who had strabismus but no significant refractive error. The most frequent refractive error was astigmatism (60%) followed by anisometropia (26%), hypermetropia (22%) and myopia (17%). Distribution of types of refractive errors in children needing glasses is summarized in Fig. 2. 93 children with amblyopia risk factors were found. Amblyopia was present in 49 children. 33 children (67%) were newly diagnosed with amblyopia because of screening.

Fifty-four children had a GSE due to referral based on unable reading on PlusoptiX. When unable readings were excluded PPV of PlusoptiX was 83%. Of the children referred because of unable readings GSE revealed normal examination in 29 (53%) and either strabismus or high refractive error beyond the instruments measurement range in 15 (27%) children. The remaining had hypermetropia (*n* = 3), myopia (n = 3), astigmatism (*n* = 2), nystagmus (*n* = 1) and ptosis (*n* = 1).

Forty-four children referred by PlusoptiX were in glasses at time of screening. When excluded from the cohort PlusoptiX would have referred 266 children (9.4%). PPV of PlusoptiX was then calculated 64%. When unable readings were excluded PPV rose to 78%.

## Discussion

This is the first report from Turkey on utilization of 4th generation PlusoptiX S12 mobile photoscreener for high volume field testing of refractive errors in schoolers. This study was conducted in Caycuma region of Zonguldak city which is a farming area and children attending rural primary schools were screened. As of 2016 1.3 million children are reported to live in rural areas of Turkey. Currently in Turkey vision screening of children with a head start in 2016 is performed by public health doctors using LEA symbols at 36–48 months of age during healthy child visits [14]. There is no state mandated school-based vision screening program.

Population-based reports from various regions in Turkey has shown the prevalence of amblyopia to range between 1.8 to 5.5% [15–19]. Studies on Istanbul and Eskisehir schoolers reported a referral rate of refractive errors to be 9 and 10.4% [16, 17]. The most frequent refractive error was astigmatism (range 5.1–14%) followed by myopia (range 3.2–6%). Incidence of hyperopia varied according to cutoff value between studies and also depending on whether cycloplegia was used. A screening study (without cycloplegia) [17] using ≥2.0 D cutoff reported an incidence of 0.6% hyperopia prevalence whereas a study using cycloplegia reported 5.9% [18] hyperopia prevalence. Distribution of the refractive errors among our study population referred by PlusoptiX is in agreement with the previously reported prevalence studies from Turkey ranking astigmatism as the most frequent.

Incidence of spectacle wear was found in some regions of Turkey to be as low as 1.7% among primary school children [19]. Our study similarly revealed a low spectacle wear of 2%. Of the amblyopic children screened 67% were newly identified during this study. Under-diagnosis of amblyopia in our cohort of schoolers was noted because the screening policy was adopted at the same time as the start of this study and the study population is naive to prior vision screening. The unable readings are promptly referred according the published guidelines on children unable to complete the protocol for automated screening. Unable screens in this study were observed in 3.6% of the study population and comprised 32% of the referrals. Only 5 was due to poor cooperation and only 2 were inconclusive. The majority was due to instrument’s inability to obtain a reading. Of the 54 children who were referred due to unable reading half had a normal examination. The issue with high unables using PlusoptiX has been reported by Kinori et al. [20] screening 3–5 years of age and Crescioni et al. [21] 8.6–15.6 years of age. The reason behind may relate to the technology utilized by digital photoscreening. Image may not process sufficiently if the eyes are not looking directly at the instrument, or the pupils may be too small when the ambient light is not dim. The ideal dimly lit environment was not present in all the classrooms during screening. Furthermore students waiting to be screened were positioned in the same room while their peer is being actively screened which may have disturbed the attention of the screened child. Based on these we recommend paying attention to pupil diameter in case of unable readings. The screener should try to dim lights in the room in case of small pupils and vice versa when the pupils are too large.

This study has several strengths. First the school-based rural area sample provides good indication of how the instrument will perform in real-world screening compared to pediatric ophthalmology patient-based sample. Our results may reflect screening performance when conducted in an environment where the room lighting is not controlled versus a clinical setting. PlusoptiX had excellent PPV (83%) when a reading was obtained however due to the 23% incidence of unable readings among referred the PPV was 69% overall.

Vision screening using visual acuity chart tests can reliably detect myopia but not hyperopia and astigmatism in school children [22]. In comparison to chart testing alone (using crowded logMAR tests), with a reported PPV of 32% [23], using plusoptiX alone in our study population has demonstrated a PPV of 69%. This suggests the cost implications of a photoscreener may be justifiable in populations with high prevalence of astigmatism and hyperopia where it will prevent numerous over referral of children with no refractive error.

Only 48% of the referred children had a GSE and maybe considered as a limitation. Also data was not presented on normal children who passed screening because they did not undergo GSE. Therefore sensitivity and specificity could not be directly determined. However one can use the amblyopia referral rate found in our study and a generally accepted amblyopia prevalence rate of 5% to calculate an estimate of sensitivity and specificity [3]. Our results would then estimate a sensitivity of 72% and a specificity of 92%.

The evidence presented here suggests that the PlusoptiX device may have a role in vision screening under the same conditions as VA assessment. Photoscreener easily operable by non-ophthalmic professionals could replace the VA assessment element of the screening thus decreasing the demand for trained personnel and also the time requirements allowing a greater number of children to be screened. In areas with no school vision screening programs the device may serve as screening tool to detect amblyopia risk factors in school age population.

## Conclusions

This field population study is first study using automated objective vision screeners in rural part of Turkey for screening for amblyopia and amplyopia risk factors. Astigmatism and hyperopia were found to be the most frequent refractive errors. Positive predictive value of 69% found suggests that the probability that the photoscreener will detect a true positive result may be higher than visual acuity testing alone. This is especially true for visual acuity measures in the presence of astigmatism and hyperopia. Screening with PlusoptiX may be viable alternative screening method for amblyopia and amblyopia risk factors for ages 5 and under as well as school aged population.

## Abbreviations

ARF: Ambyopia risk factors
D: Diopters
GSE: Gold standard eye examination
